# Reported associations between kratom and seizures: a systematic review

**DOI:** 10.3389/fphar.2025.1632835

**Published:** 2025-10-15

**Authors:** Toby Turla, Grayson Abele, My Hua, Dennis Paustenbach

**Affiliations:** ^1^ Paustenbach and Associates, Denver, CO, United States; ^2^ Paustenbach and Associates, Glendale, CA, United States; ^3^ Paustenbach and Associates, Jackson, WY, United States

**Keywords:** kratom, mitragynine, 7-hydroxymitragynine, seizures, polysubstance-use, herbal supplements

## Abstract

**Statement of purpose, innovation or hypothesis:**

Kratom, derived from the leaves of the plant *Mitragyna speciosa*, is an herbal supplement that has been used medically and recreationally for centuries, but has recently gained popularity in the United States for its analgesic and euphoric effects. The United States Food and Drug Administration (US FDA) has issued warnings against the use kratom due to potential health effects, including seizures. Seizures are one of the most commonly reported side effects associated with kratom use. The purpose of this paper is to determine whether there is a causal relationship between kratom use and seizures.

**Description of methods and materials:**

A systematic literature search was conducted to identify reviewed articles that contained information or data on kratom users experiencing seizures. Additionally, we mined adverse event data submitted to the Centers for Food Safety and Applied Nutrition’s Adverse Event Reporting System (CAERS) database and the US FDA Adverse Event Reporting System (FAERS). These databases were selected to examine consumer-reported and healthcare-reported adverse event, providing a comprehensive list of seizure-related incidents potentially associated with kratom use.

**Data and results:**

Our search yielded a total of 42 articles, but only 11 met the selection criteria for inclusion. The 11 peer-reviewed articles included for analysis consisted of case reports (n = 6), case series (n = 2), retrospective observational (n = 2), and a paper discussing a summary of adverse events reported to the FAERS database. Across the 11 publications used in our analysis, we noted a total of 20 patients who reportedly experienced seizures after using kratom. However, only one patient was quantitatively positive for Mitragynine in their urine sample, so there was minimal proof of kratom use. There was no indication that the other 19 patients had their urine subjected to mitragynine screening which would have confirmed their use of kratom and internal metabolism after intake. Furthermore, the FAERS dashboard, only 22 of 481 kratom-related reports involved seizure (4.5%); the CAERS database had 18 reported seizures amongst 221,178 kratom-related incidents. Information regarding the quantity of kratom consumption or confirmed use of kratom was not available in any of the datasets reviewed.

**Interpretation, conclusion or significance:**

Currently, the available literature primarily consists of a limited number of studies, including case reports and case series, which document self-reported kratom use and provide minimal quantitative toxicological data on mitragynine. Based on our narrative review, we conclude that there is insufficient evidence to suggest that kratom causes seizures, primarily due to the absence of a quantitative dose-response in toxicology reports, a incomplete medical records and because seizures occur relatively frequently in the general population.

## Highlights

• From case reports (N = 6) and case series (N = 14), we identified a total of 20 individuals who have reported to have experienced seizures following kratom use
• Due to inconsistent medical records, complex medical histories, and the absence of blood concentrations of mitragynine (a marker of kratom use), case reports and series should not be regarded as robust data correlating the effects between kratom and seizures.
• Federally available data from the U.S. FDA does not include information on kratom dosage, frequency, and duration of use, nor does it provide medical histories or blood mitragynine concentrations. Therefore, it was premature for the FDA to alert the public on kratom-induced seizures when there was very limited and compromised evidence.
• The lack of a known mechanism by which mitragynine induces seizures suggests that there is currently no scientific evidence to support a direct causal relationship between kratom use and the occurrence of seizures.

## 1 Introduction

Kratom, derived from the Southeast Asian tree *Mitragyna speciosa*, was introduced into the United States commercially in the 1980s and gained popularity from the mid-2000’s onward (Henningfield et al., 2018; Kruegel and Grundmann, 2018; Veltri and Grundmann, 2019). Seizure reports are sparse and limited to non-recurring cases in Southeast Asia (Halim et al., 2021). In contrast, Western literature has documented focal and generalized seizures in kratom-users, sometimes with severe outcomes (Boyer et al., 2008; Nelsen et al., 2010; Tatum et al., 2018; Hughes, 2019; Afzal et al., 2020; Cumpston et al., 2018; Burke et al., 2021).

Kratom has been used for both recreational and alternative medicinal purposes for centuries in Southeast Asia, including combating fatigue from manual labor, managing chronic pain, addressing PTSD, and reducing anxiety (Grundmann, 2017; Singh et al., 2017; Coe et al., 2019; Eastlack et al., 2020). The plant has been known by various local names depending on the region in Southeast Asia, such as “biak biak” in Malaysia or “thom” in Thailand (Cinosi et al., 2015; Gong et al., 2012).

Traditionally consumed as tea or dried leaves, modern kratom products include powders, capsules, and liquid extracts (Singh et al., 2016). Rural communities such as farmers, laborers, and fishermen used kratom as a stimulant to alleviate fatigue by chewing the leaves raw or drying them for later consumption in teas or chewing (Cinosi et al., 2015; Grundmann, 2017; Singh et al., 2017). Although not a hallucinogen, kratom’s euphoric properties also motivated its use, and it was sometimes offered as a gift to gods (Swogger et al., 2015; Saingam et al., 2016).

Mitragynine, an indole-based alkaloid with the empirical formula C_23_H_30_N_2_O_4_ and a molar mass of 398.5 g/mol, is the primary active compound in kratom. It binds to µ-opioid receptors, inducing euphoria, reducing anxiety, and alleviating pain (Hassan et al., 2013); however, it is not an opioid. In humans, mitragynine is metabolized into 7-hydroxymitragynine by the CYP3A4 enzyme. 7-hydroxymitragynine has a higher binding affinity to µ-opioid receptors despite being present in smaller quantities in kratom (Chakraborty et al., 2021; Obeng et al., 2021; Mongar et al., 2024).

Medicinally, kratom has been used to treat a wide range of conditions, including substance abuse disorders, headaches, toothaches, cough, fever, sleeplessness, pain, hypertension, diabetes, and anxiety (Gong et al., 2012; Grundmann, 2017; Singh et al., 2017; Eastlack et al., 2020; Ahmad et al., 2022). The analgesic effects of kratom are due to mitragynine and 7-hydroxymitragynine, which act as partial agonists at µ-opioid receptors (MOR) and interact with kappa-opioid receptors (KOR) and delta-opioid receptors (DOR) in the brain and spinal cord (Annuar et al., 2024). These alkaloids also inhibit neurotransmitter release by blocking calcium channels on neurons, further enhancing their pain-relieving properties (Eastlack et al., 2020).

Though traditionally used in Southeast Asia, kratom is thought to have abuse potential and adverse effects, leading to bans in countries like Denmark, Singapore, and Malaysia (Veltri and Grundmann, 2019; Charoenratana et al., 2021; Webber, 2022). In Denmark, kratom has been a controlled substance and officially illegal as of 2009. Singaporean legislation bans the possession and sale of kratom and classifies kratom and its alkaloids as Class A controlled drugs (Charoenratana et al., 2021). The use of kratom in Malaysia is prohibited under the Poisons Act of 1952, but due to kratom trees growing naturally, it is still commonly used (Veltri and Grundmann, 2019).

A 2024 informational resource published by the United States Food & Drug Administration (U.S. FDA) highlighted the use of kratom in the United States, noting:

“*in rare cases, deaths have been associated with kratom use, as confirmed by medical examiners or toxicology reports (U.S. Food and Drug Administration, 2024b)*”.

However, in these cases, kratom was usually used in combination with other drugs, and the contribution of kratom to the deaths is unclear (U.S. Food and Drug Administration, 2024b).

Based on adverse event reports, the FDA has previously warned that kratom may cause seizures (U.S. Food and Drug Administration, 2025a). However, its recent focus has shifted specifically to products with 7-hydroxymitragynine (U.S. Food and Drug Administration, 2023b; U.S. Food and Drug Administration, 2025b). The FDA cautioned against its use due to the potential for serious adverse events, including toxicity, seizures, and substance use disorder (SUD) (U.S. Food and Drug Administration, 2024b). In May 2021, the U.S. FDA announced its intention to seize an adulterated dietary supplement containing kratom (U.S. Food and Drug Administration, 2021).

Self-reported side-effects following kratom consumption have included neurological effects such as agitation, drowsiness, tremor, ataxia, headache, syncope, slurred speech, and seizures. Seizures have been reported after kratom use alone, with an incidence of 6.1%–9.6% (Eggleston et al., 2019; Post et al., 2019). Documentation of seizures following kratom have been reported to be as high as 17.5% of cases (Trakulsrichai et al., 2013). Because of kratom’s documented neurological effects, specifically seizures in evaluations of kratom and its health effects, it seemed timely to better understand its safety profile.

To assess the association between kratom usage and seizures in humans, we conducted a systematic review. We analyzed demographic use data from the CAERS and FAERS databases.

## 2 Methods

We conducted a targeted literature review using PubMed with the Boolean search string “kratom OR mitragynine OR 7-hydroxymitragynine AND (seizure OR epilepsy OR convuls*)”. The asterisk (*) was used to capture variations such as convulsion(s), convulsed, and convulsing. This systematic review yielded 42 studies. We reviewed all 42 studies that attributed seizures to kratom use and identified two additional case reports through manual search methods that were not captured by the initial query.

We filtered the literature using the following inclusion criteria: (1) published in peer-reviewed journals; (2) discussed seizure occurrence attributed or chronologically relevant to kratom use; (3) reported kratom use in various forms (e.g., tea, pills, powder); and its alkaloid derivatives (mitragynine and 7-hydroxymitragynine); (4) discussed Mitragyna speciosa leaves as the primary ingredient in consumed kratom. Studies were excluded if they did not discuss seizure adverse effects in patients using kratom or were not written in English. We identified a total of 11 peer-reviewed articles from case reports, case series, and retrospective observational studies that matched the inclusion criteria. The initial review identified 42 articles, but 31 were excluded for not meeting the inclusion criteria; this was primarily because they did not examine seizure outcomes directly attributed to kratom use. For example, Spungen et al. (2024) described a seizure in an infant only indirectly exposed via maternal kratom use, and Noe et al. (2024) reported rhabdomyolysis due to benzodiazepine misuse, with neither study aligning with this review’s focus.

We appraised each case report using the Joanna Briggs Institute (JBI) Checklist for Case Reports. The completed assessments are provided in the Supplemental Materials. This appraisal was performed to evaluate the potential bias and variability in the reporting of cases.

To explore seizure-related data submitted by healthcare providers and consumers who reported seizure effects related to kratom use, we accessed the FDA Center for Food Safety and Applied Nutrition (CFSAN) Adverse Event Reporting System (CAERS) website and downloaded reports from January 2004 to July 2024. The CAERS database collected adverse events, and product complains related to dietary supplements, cosmetics, and food (U.S. Food and Drug Administration, 2024a). We filtered the CAERS dataset using the search terms “kratom” and “mitragynine” and reviewed demographic data. We mined the FDA’s Adverse Event Reporting System (FAERS), a database previously examined by Li et al. (2023), which monitored adverse reactions in medications and therapeutic biologicals. In the FAERS dataset, we utilized the terms “7-hydroxymitragynine” and “herbals\mitragynine” to identify kratom-related reports. We reviewed cases of seizure-related incidents associated with kratom products.

## 3 Results

The 11 peer-reviewed articles that were selected included case reports (n = 6), case series (n = 2), retrospective observational (n = 2), and a summary of adverse effects reported to the FDA (n = 1).

From the six case reports and two case series we filtered in our systematic review, a total of 20 patients reportedly experienced seizures associated with kratom use. Most of the patients were males (n = 19, 95%). The ages of the 20 patients ranged from 19 to 64 years of age, with an average age of 29 years. These patients presented at the hospital with generalized tonic-clonic seizures (n = 14), focal-to-bilateral (n = 5), and/or bilateral seizures (n = 1). One patient experienced a total of six seizures, which chronologically included two generalized tonic-clonic seizures, a focal to bilateral generalized tonic-clonic (GTC) seizure, a focal seizure, and another two GTC seizures (Tatum et al., 2018).

Pre-existing medical conditions included anxiety (n = 2), chronic pain (n = 2), depression (n = 1), cancer (n = 1), attention deficit hyperactivity disorder (ADHD) (n = 2), prior opioid abuse (n = 3), a post meningioma resection (n = 1), a left temporal cavernoma with resection (n = 1), and a soft tissue hematoma (n = 1). Five patients used kratom in combination with other substances such as opioids (n = 3), antiepileptic medications (n = 1), and benzodiazepines (n = 1). Polysubstance use was reported in ten cases, while nine patients were documented as having a mono-substance use history. One case did not provide information about additional substance use. A summary of the case characteristics and patient demographics is presented in Table 1. Table 2 provides detailed information specifically related to mitragynine exposure and associated patient data.

**TABLE 1:** Case characteristics and patient demographics.

Patient #	Age	Gender	Reported neurological activity following kratom use	Documented polysubstance use	Relevant previous medical history	Results of toxicology screening	Authors
1	43	Male	GTC	Yes	Chronic pain from thoracic outlet syndrome managed by hydromorphone	Modafinil (urine)	Boyer et al. (2008)
2	64	Male	Generalized seizure	Yes	Colostomy repair, chronic pain, depression managed by amitriptyline, oxycodone, kratom	Cannabinoids (urine), tricyclic antidepressant (urine), oxycodone (urine), Mitragynine (urine: 167 ± 15 ng/mL)	Nelsen et al. (2010)
3	19	Male	Tonic-clonic seizure	Yes	Attention deficit hyperactivity disorder managed by lisdexamfetamine dimesylate	N/A^a^	Tatum et al. (2018)
4	27	Male	Generalized seizure	Yes	Asperger Syndrome, bipolar disorder, and substance abuse	Quetiapine (blood: 12,000 ng/mL), Valproic acid (blood: 8.8 mcg/mL)	Hughes (2019)
5	27	Male	Tonic-clonic seizure	Yes, diphenhydramine	Anxiety, ADHD, benzodiazepine-use, and opioid use disorder	N/A^a^	Afzal et al. (2020)
6	45	Male	Generalized seizure	N/A^a^	N/A^a^	N/A^a^	Cumpston et al. (2018)
7	25	Male	Focal to bilateral tonic-clonic	Yes	N/A^a^	Opioids (urine: 44 ng/mL)	Halim et al. (2021)
8	20	Male	GTC	No	N/A^a^	Negative^b^	
9	27	Male	Focal to bilateral tonic-clonic	No	N/A^a^	Negative^b^	
10	20	Male	GTC	No	N/A^a^	Negative^b^	
11	22	Male	GTC	No	N/A^a^	Negative^b^	
12	25	Male	Focal to bilateral tonic-clonic	Yes	N/A^a^	Amphetamine-type stimulant (urine: 631 ng/mL) and opioids (urine: 87 ng/mL)	
13	29	Male	Focal to bilateral tonic-clonic	No	N/A^a^	Negative^b^	
14	21	Male	GTC	No	N/A^a^	Negative^b^	
15	17	Male	GTC	No	N/A^a^	Negative^b^	
16	22	Male	GTC	No	N/A^a^	Negative^b^	
17	25	Male	GTC	No	N/A^a^	Negative^b^	
18	49	Female	Focal to bilateral tonic-clonic epilepsy	Yes, eslicarbazepine and lacosamide	Post-meningioma resection 3 years prior; 6 seizures in 1 month	Lacosamide (urine: 2.3 μg/mL), Eslicarbazepine (urine)	Burke et al. (2021)
19	37	Male	Bilateral-tonic clonic epilepsy	Yes, levetiracetam and opioids	Opioid-use disorder, left temporal cavernoma with resection	Tetrahydrocannabino (urine), benzodiazepine (urine), levetiracetam (urine: 20.7 μg/mL)	
20	24	Male	Generalized tonic-clonic epilepsy	Yes, opioids	Opioid-use disorder, stress and anxiety	N/A^a^	

^a^ No additional substances of concern were recorded by the author.

^b^ Urine toxicology tested for morphine, cannabis, benzodiazepines, barbiturates, phencyclidine, and amphetamines.

GTC: generalized tonic clonic.

**TABLE 2:** Mitragynine specific information.

Patient #	Mitragynine specific concentrations from drug screening	Volume of kratom consumed	Frequency	Duration	Authors
1	Not quantified	N/A; consumed kratom via tea-drinking	Daily (4x day)	3.5 years consistently to avoid hydromorphone-use and manage pain	Boyer et al. (2008)
2	Mitragynine (urine: 167 ± 15 ng/mL)	N/A; consumed kratom via Datura tea-drinking 30 min the seizure	NA	Regular-user of kratom for pain, self-reported	Nelsen et al. (2010)
3	NQ	N/A: “several pills per day”	Daily	NA: “several months prior”	Tatum et al. (2018)
4	NQ	N/A	N/A	N/A	Hughes (2019)
5	NQ	3–4 bottles of 8 mL	Daily	1.5 years	Afzal et al. (2020)
6	NQ	N/A	N/A	Intermittently ingested kratom for 10 months	Cumpston et al. (2018)
7	Urine mitragynine positive^a^	500–1,000 mL	≥8 per month	60 months	Halim et al. (2021)
8	Urine mitragynine positive^a^	<200 mL	≥8 per month	3 months	
9	Urine mitragynine positive^a^	>1,000 mL	≥8 per month	84 months	
10	Urine mitragynine positive^a^	200–500 mL	≥8 per month	12 months	
11	Urine mitragynine positive^a^	200–500 mL	≤3 per month	5 months	
12	Urine mitragynine positive^a^	>1,000 mL	≥8 per month	84 months	
13	Urine mitragynine positive^a^	200–500 mL	≤3 per month	48 months	
14	Urine mitragynine positive^a^	200–500 mL	≥8 per month	24 months	
15	Urine mitragynine positive^a^	200–500 mL	≥8 per month	24 months	
16	Urine mitragynine positive^a^	>1,000	≥8 per month	60 months	
17	Urine mitragynine positive^a^	200–500 mL	≥8 per month	24 months	
18	NQ	NQ	Daily	NQ	Burke et al. (2021)
19	NQ	NQ	Daily	NQ	
20	NQ	NQ	NQ	5 months total	

^a^ Quantitative values of mitragynine from urine samples were not reported.

NQ: not quantified.

### 3.1 Case reports

Six case reports representing six patients were identified. Five case reports did not provide relevant kratom use parameters, such as dose, frequency, and duration of use (Boyer et al., 2008; Nelsen et al., 2010; Cumpston et al., 2018; Tatum et al., 2018; Hughes, 2019). Among these, Afzal et al. (2020) was the only case report to present detailed information on these parameters.

Toxicological tests were positive in three case reports for other relevant substances including modafinil, cannabinoids, tricyclic antidepressants, oxycodone, quetiapine, and valproic acid (Boyer et al., 2008; Nelsen et al., 2010; Hughes, 2019). Hughes (2019) was the only case report in our review where death occurred, and a postmortem autopsy suggested seizure-like activity. Hughes (2019) was the only case report that provided quantitative toxicology values (quetiapine (12,000 ng/mL) and valproic acid (8.8 mcg/mL)).

Nelsen et al. (2010) was the only case report to provide mitragynine-specific concentrations, reporting levels of 167 ± 15 ng/mL in urine. However, the researchers did not specify the dose or frequency of kratom consumption, despite indicating regular use.

### 3.2 Case series

A total of two case series were identified involving kratom use and seizure occurrence (Burke et al., 2021; Halim et al., 2021).

Burke et al. (2021) documented seizure occurrences in three patients following kratom consumption, all of whom had complex medical histories and showed signs of polysubstance use. Burke et al. (2021) did not provide specific details about kratom dose, frequency, and duration.

Halim et al. (2021) reported kratom-specific consumption, frequency, and duration for 11 patients. However, researchers did not provide quantitative biological samples of mitragynine concentrations in patients. The study authors noted that 8 of the 11 sources of kratom consumed by patients experiencing seizures were from a “local supplier”. Toxicological tests were qualitatively positive for the 11 patients. The time interval between ingesting kratom and seizure onset ranged from 10 min to 72 h (Halim et al., 2021). Halim et al. (2021) screened for morphine, cannabis, benzodiazepines, barbiturates, phencyclidine, and amphetamines.

### 3.3 Retrospective observational studies

Two retrospective observational studies, one using the United States National Poison Data System (US NPDS) and the other using the Ramathibodi Poison Control Center of Thailand, contained data on adverse health effects associated with kratom use. However, kratom-specific intake details were not documented in either study (Trakulsrichai et al., 2013; Eggleston et al., 2019).

Eggleston et al. (2019) identified a total of 2,312 kratom exposures reported to the US NPDS. Of these, 935 were single-substance exposures to kratom, with 56.5% of those reports indicating consumption through tablets, capsules, or powder. Severe adverse events included agitation (18.6%), tachycardia (16.9%), drowsiness (13.6%), vomiting (11.2%), and seizures (6.1%). However, it was unclear whether this 6.1% referred to the 935 single-substance exposures or the total of 2,312 kratom exposures (Eggleston et al., 2019).

Trakulsrichai et al. (2013) analyzed kratom exposure cases from Thailand’s toxic surveillance system at the Ramathibodi Poison Center (RPC) to assess clinical characteristics of kratom-exposed cases suspected of developing poisoning and withdrawal. Of the 52 kratom exposures, 40 involved poisoning and 12 involved withdrawals. Among the 40 kratom poisoning cases, seven patients (17.5%) reported seizures. Substances such as codeine, amphetamine, Cola beverage, and diphenhydramine were ingested in 17 cases, but the authors did not specify how many of these polysubstance use reports were attributed to kratom use. Similar to (Eggleston et al., 2019), Trakulsrichai et al. (2013) did not report kratom-specific information such as dose, frequency, or duration of use.

### 3.3 A summary of adverse reactions in the U.S. FDA: FAERS and CAERS

#### 3.3.1 FAERS

A study by Li et al. (2023) summarized adverse effects from 489 kratom-related incidents reported to the US FDA Adverse Event Reporting System (FAERS) between October 2012 and September 2021. The authors used preferred terms from the Medical Dictionary for Regulatory Activities (MedDRA) to define these adverse reactions.

Of the 489 kratom-related adverse reports, only 22 patients (4.5%) reported seizures. Li et al. (2023) calculated significant signals of seizures (log OE ratio 3.15, 95% credibility interval 2.50–3.60) and generalized tonic-clonic seizure (log OE ratio 2.5, 95% credibility interval 1.05–3.59). The researchers cited studies on seizure instances from kratom use by Tatum et al. (2018), Afzal et al. (2020), and Burke et al. (2021). The authors suggested the adrenergic and stimulant effects of kratom may explain the seizures, although the mechanism of action remains unclear (Li et al., 2023).

As of January 2025, an investigation into the FAERS public dashboard documented 29,661,136 adverse events. Of the total events reported, 1,255 were related to “7-hydroxymitragynine” and/or “herbals\mitragynine”.

We further investigated “Case Count by Reaction”, which reported signs and symptoms following kratom use. Of the 1,255 adverse reports, “seizures” had 74 hits. “Toxicity to Various Agents” (N = 281), “Drug Abuse” (N = 171), and “Drug Interaction” (N = 162) were the top three adverse reactions counted by FAERS. The evaluation of the FAERS dataset was initially conducted and summarized by Li et al. (2023). They observed duplications which may accidentally occur in adverse drug reaction reporting, and adjusted for them (Hauben et al., 2007).

#### 3.3.2 CAERS

In addition to the FAERS database, we examined data from the Center for Food Safety and Applied Nutrition Adverse Event Report System (CAERS). Upon downloading the CAERS dataset, we identified 221,178 cases. We filtered for “kratom” and “mitragynine,” which yielded 709 hits. The earliest recorded date from this dataset was on or about June 2017.

From the 709 results, we further filtered the data for seizure occurrences, which resulted in 18 reports. Of the 18 reports, 16 (88.89%) were male and 2 (11.11%) were female. The average age of the patients was 35.86, with ages ranging from 22 to 81 years. Only 1 of the 18 cases reported seizure as the only symptom exhibited by the patient, whereas the other 17 cases had other symptoms. These symptoms included nervous system lesion, cardiac arrest, pain, withdrawal, overdose, and psychotic disorder. The CAERS dataset dichotomized products as either “suspect” or “concomitant”. All 18 reports of seizure occurrence and kratom use were listed as “suspected”.

## 4 Discussion

The neurological health effects from kratom and its alkaloids, mitragynine and 7-hydroxymitragynine, are understudied. Previous data suggested an estimated kratom user population of 10–16 million in the United States (Henningfield et al., 2019; Heywood et al., 2024). The growing number of kratom users has prompted healthcare providers to be aware of its use, especially when consumed in tandem with other medications (Striley et al., 2022; McCurdy et al., 2024).

The goal of the current review was to identify and examine relevant papers describing the co-occurrence of seizures following kratom use, as well as to determine whether a dose-response relationship exists. No dose relationship was identified from the existing case series, case reports, and available federal adverse events reporting databases. Seizures reported in Southeast Asia and the West could exhibit differences in their severity due to the variability of mitragynine in kratom products. The formulations of kratom consumed in Southeast Asia are recognized to typically be different from the products used in the United States. Key demographics are discussed below, along with limitations of the cited studies, emphasizing gaps in the current data and the need to research potential risk factors for seizures and kratom use.

### 4.1 Study demographics

Most patients experiencing seizures following kratom use were male, and all were between the ages of 19 and 64 years. Garcia-Romeu et al. (2020) performed a cross-sectional study that showed the mean age of reported kratom users was 40 years old, and that they were predominantly males. Similar demographics were identified in Grundmann (2017), where kratom use occurred primarily among White men aged 31–50 years. The origins of published case reports and case series include the United States and Thailand. Of the 20 patients, only two were identified as Caucasian, while the racial background of the remaining 18 was unspecified. Although these demographic characteristics provide a summary of the available literature, they reflect a limitation in reports concerning adverse events associated with kratom use among men and women. The US CAERS dataset was the only federally available database that provided age and gender demographics of kratom use and adverse effects such as seizures. A proposed research question is to determine whether certain racial demographics are at greater risk for seizures following kratom use.

### 4.2 Strengths

Although case reports, case series, and federal databases have inherent limitations, the studies in this discussion also possess notable insights. Case reports often provide in depth details, clinical observations, timelines, and proposed hypotheses for patient outcomes. The researchers provide thorough documentation of patient medical histories, including neurological, psychiatric, and medication histories (Boyer et al., 2008; Nelsen et al., 2010; Cumpston et al., 2018; Tatum et al., 2018; Hughes, 2019; Afzal et al., 2020). Afzal et al. (2020) discussed factors such as ADHD and anxiety, which may predispose individuals to seizures. Two studies propose potential mechanisms by which kratom and its alkaloids may induce seizures (Hughes, 2019; Afzal et al., 2020). Several authors also acknowledged the limitations due to the lack of quantitative mitragynine concentrations and the anecdotal nature of seizure reports (Cumpston et al., 2018; Tatum et al., 2018).

Aggregated patient data provided a broader overview of kratom-related health effects. Halim et al. (2021) excluded patients with prior brain injury or neurological conditions, which reduced confounding variables. Their comprehensive overview of urine toxicology test screening for several substances revealed opioid use (44 ng/mL and 87 ng/mL) and amphetamine-type stimulant use (631 ng/mL) in three patients. Moreover, this study considers kratom source (e.g., local supplier versus self-prepared), providing context for understanding variability in alkaloid concentrations and potential contaminants (Halim et al., 2021).

### 4.3 Limitations

Literature shortcomings prevented us from establishing a causal link between kratom use and seizures. These limitations included (1) a lack of standardized blood or urine testing for kratom-related alkaloids, (2) confounding factors such as polysubstance use, (3) uncomprehensive medical histories, (4) missing use parameters (i.e., dose, frequency, and duration of kratom use), (5) study types relying on anecdotal self-reports and overall small sample size, and (6) variability of kratom product formulation and product concentration. Based on the available data, construction of a dose-response relationship is not possible. This limitation arises primarily from kratom’s relatively recent emergence in the United States, which means there is a lack of systematic data collection.

#### 4.3.1 A lack of standardized testing for kratom alkaloids

Of the 20 patients who had seizures after kratom use, only one underwent urine testing for mitragynine (167 ± 15 ng/mL) (Nelsen et al., 2010). In the study by Halim et al. (2021), eleven patients were reported to be qualitatively positive following urine toxicology. No quantitative data was presented.

Interestingly, the retrospective observational in Eggleston et al. (2019) provided postmortem blood concentrations in decedents (N = 4) who used kratom but not in those who experienced seizures. Mitragynine concentrations in postmortem blood ranged from 5.4 to 11,000 ng/mL, complicating definitive conclusions about kratom and mortality (Papsun et al., 2023). The Ramathibodi Poison Control Center did not provide the results of drug testing in the 52 patients, but reported that in 40 of these cases, acute kratom poisoning was noted. Of the 40 patients who suffered acute kratom poisoning, 7 (17.5%) reported seizures.

The lack of toxicological testing renders the term “poisoning” arbitrary. FAERS and CAERS lacked kratom-specific information regarding kratom-specific products or drug testing data, highlighting the inconsistencies in reporting kratom-related adverse events. Sources of biological specimens include urine, blood, saliva, hair, and sweat (Hadland and Levy, 2016). Blood sampling is a widely used laboratory protocol to depict recent use of ingested substances, but it is more invasive than urine samples. Substances, like mitragynine, are metabolically converted into more water-soluble forms, such as 7-hydroxymitragynine, to promote urinary excretion; therefore, serum blood samples are widely accepted as an ideal method for assessing moment-to-moment substance levels (Ramanathan et al., 2015; Suhaimi et al., 2016; Mukherji et al., 2023).

Blood testing allows for precise levels of drugs to be assessed in clinical investigations (McNeil et al., 2023). Blood concentrations of mitragynine in kratom users would provide a direct measure of pharmacological effects compared to urine concentrations. Urine concentrations primarily provide more information on qualitative presence, whereas blood concentrations are more quantitative. This distinction provides a rationale for more comprehensive toxicological panels, particularly for blood mitragynine, in patients experiencing adverse effects such as seizures. Integrating quantitative toxicology data with detailed clinical observations, such as those in case reports and series, would help identify a dose-response relationship between kratom use and seizures. Mitragynine does not routinely appear on standard toxicology panels, which further restricts the availability of quantitative exposure data.

#### 4.3.2 Polysubstance use as a confounding factor

Polysubstance use introduces unpredictable variables that make it difficult to identify the effects of a single substance on an outcome of interest. High performance liquid chromatography of subclavian blood quantitatively identified Valproic acid at 8.8 mcg/mL and Quetipiane at 12,000 ng/mL. Quetiapine is an antipsychotic medication and was found to be in the lethal dose range of 1.8–20 mg/L (Molina and Hargrove, 2019). Urine drug screening tests performed by Halim et al. (2021) tested eleven patients for morphine, cannabis, benzodiazepines, barbiturates, phencyclidines (PCP), and amphetamines. Halim et al. (2021) reported that three patients had positive drug screening results. Two patients tested positive for opioids (at concentrations of 44 ng/mL and 87 ng/mL), and one patient tested positive for 631 ng/mL of amphetamine-type stimulants. Though the type of opioid or amphetamine was not specified, these agents are known to cause seizures in humans (Stevens et al., 2013; Regina et al., 2025). While the remaining seven patients were reported to be negative for other substances, four had consumed kratom mixed with antihistamine diphenhydramine, which was not assessed on the drug screening panel.

Oversight of antihistamines from the toxicology panel presented a potential gap in information, as unconfirmed polysubstance use could influence seizure risk (U.S. Food and Drug Administration, 2020; Kim et al., 2021). Additionally, cocaine, another known seizure-inducing substance, was notably absent from the toxicology report (Richards and Laurin, 2023). The Ramathibodi Poison Control Center documented 17 polysubstance cases in the 52 kratom-related incidents but did not indicate if seizures occurred or if additional substances were co-administered. Furthermore, the FAERS and CAERS databases relied on self-reported information and did not provide detailed polysubstance histories. The presence of additional substances, quantitatively or qualitatively confirmed, complicates the assessment of kratom’s role in clinical outcomes, as drug interactions with kratom are understudied.

Comprehensive toxicology panels provide quantitative information on kratom’s alkaloids, which would be beneficial in determining the relationship between kratom use and seizure occurrence. There is no current research which clearly shows that polysubstance use involving kratom can increase the risk of seizures; further research is warranted in this area. It has been documented that the involvement of other compounds may increase the risk of seizures (Alldredge et al., 1989; Wolfe et al., 2019).

#### 4.3.3 Confounding factors: complex medical histories

All case reports indicated complex medical histories among cases, including bipolar disorder, seizure disorders, ADHD, anxiety, benzodiazepine use disorder, opioid use disorder, chronic pain, thoracic outlet syndrome, hydromorphone use disorder, Asperger Syndrome, substance abuse disorder, and inconsistent adherence to antiepileptic medications (Boyer et al., 2008; Nelsen et al., 2010; Tatum et al., 2018; Hughes, 2019; Afzal et al., 2020). Cumpston et al. (2018) did not provide a medical history for the patient; however, a head CT revealed a nasal bone fracture and soft tissue hematoma. The location of the soft tissue hematoma was not provided. Without a comprehensive overview of the patient’s medical history and location of the hematoma, it is not possible to attribute the seizures to kratom use alone, as preexisting risk for seizures could not be accounted for. In Burke et al. (2021), histories of neuropsychiatric illnesses were prevalent and included epilepsy, meningiomas, cavernoma malformation, and opioid use disorder. Retrospective studies and available federal datasets did not provide preexisting information are important in assessing the relationship between kratom use and seizure occurrence (Trakulsrichai et al., 2013; Eggleston et al., 2019; Li et al., 2023; U.S. Food and Drug Administration, 2024a).

Comprehensive medical histories are important because they provide clinicians with an overview of past and present conditions to make a proper diagnosis. Previous medical histories allowed healthcare providers to identify risk factors and potential contraindications in treatments. In the context of investigating the relationship kratom and seizures, complex medical histories, particularly those involving neuropsychiatric conditions, can obscure the causal relationship. Conditions such as substance use disorder and epilepsy, both of which are documented contributors to seizure activity must be assessed.

#### 4.3.4 Lack of dose, frequency, and duration data for kratom use

No case report provided a complete documentation of kratom dose, frequency, and duration (Boyer et al., 2008; Nelsen et al., 2010; Cumpston et al., 2018; Tatum et al., 2018; Hughes, 2019; Afzal et al., 2020). Halim et al. (2021) was the only case series that reported kratom dose, frequency, and duration. Trakulsrichai et al. (2013) and Eggleston et al. (2019) did not provide kratom consumption details. While the FAERS and CAERS datasets provided the brand name of the product containing kratom or kratom alkaloids, they did not provide kratom consumption specifics. Missing information about kratom consumption patterns prevents researchers from establishing a dose-response relationship.

#### 4.3.5 Limitations of anecdotal self-reports and small sample sizes

Case reports are reported in (Boyer et al., 2008; Nelsen et al., 2010; Cumpston et al., 2018; Tatum et al., 2018; Hughes, 2019; Afzal et al., 2020) Burke et al. (2021) and Halim et al. (2021) relied on anecdotal or self-reported data, lacked control groups, and have small sample sizes, limiting generalizability (Hajian-Tilaki, 2011; Nissen and Wynn, 2014). Without a control group for comparison, or a group of unexposed subjects with characteristics, or a group of unexpected subjects with similar characteristics to cases, the incidence of adverse events at a population level cannot be determined (Kestenbaum, 2019). Retrospective studies (Trakulsrichai et al., 2013; Eggleston et al., 2019) and federally available databases (U.S. Food and Drug Administration, 2023a; U.S. Food and Drug Administration, 2024a) are prone to missing data, reporting bias, and duplication, further complicating interpretation. Overall, study designs and small sample sizes restricted the ability to establish an association, let alone a causal relationship between kratom use and seizure occurrence.

#### 4.3.6 Variability of kratom product formulation and product concentration

Variability in product formulation can contribute to inconsistencies in the concentration of mitragynine and 7-hydroxymitragynine. As seen in Tables 1, 2, mitragynine-specific information is documented across teas, liquid extracts, and pills. Halim et al. (2021) indicated subjects acquired premade kratom-containing products from local suppliers on prepared kratom concoctions themselves. Eight of the eleven patients reporting seizures following kratom use sourced their kratom from a local supplier, which may indicate the presence of an adulterated substance to enhance kratom’s effect or contamination with a foreign agent. The concentrations of kratom alkaloids vary depending on the method of preparation, including teas, leaves, powders, pills, and extracts (Heywood et al., 2024).

The type of kratom preparation is not specified in the FAERS and CAERS databases; however, brand specifics were provided (Li et al., 2023; U.S. Food and Drug Administration, 2023a; U.S. Food and Drug Administration, 2024a). Although clinical trials evaluating kratom are limited, recent pharmacokinetic data reported by Huestis et al. (2024) demonstrated that single and multiple doses of kratom capsules resulted in higher ratios of the metabolite 7-hydroxymitragynine relative to its parent compound, mitragynine, at lower doses in patient blood. These findings show the importance of considering CYP3A4 enzyme activity, potential enzyme saturation at higher doses, and product concentration variability when interpreting pharmacokinetic data of kratom’s various mediums.

## 5 Conclusion

Kratom has become popular in the United States, with users often consuming the herbal through teas. Mitragynine concentrations in kratom products are typically less than 15–30 mg/g and oral doses of under 20 mg/kg-bw-day are not predicted to result in adverse health effects (Heywood et al., 2024). Several case studies and series have reported seizures following kratom use (Boyer et al., 2008). However, these reports often lack specific details such as dose, frequency, and duration of use, which are crucial for understanding the potential health effects of kratom. Additionally, the absence of blood mitragynine concentrations in these studies complicates the establishment of a clear connection between kratom use and seizure occurrence.

The relationship between kratom and seizures is obscured by confounding factors. Many patients in the case series and case reports have complex medical histories including opioid disorder and preexisting neuropsychiatric illnesses. Polysubstance use was also commonly noted, raising questions about potential interaction between kratom and other drugs. Federal databases such as CAERS and FAERS lack detailed information on kratom dosage, particularly in seizure cases, Given the consistent lack of kratom use behaviors and incomplete or missing medical histories, the relationship between kratom use and seizures is not clear. To the best of our knowledge, this paper represents the first comprehensive review of reported kratom-associated seizures, synthesizing the data from case reports, case series, and public datasets.

Improved patient data collection and clinical research shows enhance the quality of future reports on kratom. Whenever possible, blood mitragynine levels should be reported alongside detailed medical histories, so that the potential dose-relationship between mitragynine concentrations and seizures may be evaluated. While papers like Huestis et al. (2024) evaluated mitragynine and 7-hydroxymitragynine pharmacokinetics in single and multiple doses in humans, we recommend robust clinical studies to examine kratom’s health effects on several different endpoints. Specifically, these studies must highlight the mitragynine dose and occurrence of seizures, and whether kratom could result in seizures at the onset of its use or later. Additional research should evaluate kratom’s therapeutic potential, particularly for those seeking unconventional medical treatments, as well as any risks.

## Data Availability

Publicly available datasets were analyzed in this study. This data can be found here: https://fis.fda.gov/sense/app/95239e26-e0be-42d9-a960-9a5f7f1c25ee/sheet/7a47a261-d58b-4203-a8aa-6d3021737452/state/analysis, https://www.fda.gov/food/compliance-enforcement-food/human-foods-complaint-system-hfcs.
